# Recent trends in hormone therapy utilization and breast cancer incidence rates in the high incidence population of Marin County, California

**DOI:** 10.1186/1471-2458-10-228

**Published:** 2010-04-30

**Authors:** Rochelle R Ereman, Lee Ann Prebil, Mary Mockus, Kathy Koblick, Fern Orenstein, Christopher Benz, Christina A Clarke

**Affiliations:** 1 County of Marin, Department of Health and Human Services, 20 North San Pedro Road, San Rafael, CA 94903, USA; 2 Kaiser Permanente Medical Group, 99 Monticello Rd, San Rafael, CA 94903, USA; 3 Zero Breast Cancer, 4340 Redwood Hwy, Suite C400, San Rafael, CA 94903, USA; 4 Buck Institute for Age Research, 8001 Redwood Blvd Novato, CA 94945, USA; 5 Cancer Prevention Institute of California, 2201 Walnut Ave., Suite 300 Fremont, CA, 94538, USA

## Abstract

**Background:**

Recent declines in invasive breast cancer have been reported in the US, with many studies linking these declines to reductions in the use of combination estrogen/progestin hormone therapy (EPHT). We evaluated the changing use of postmenopausal hormone therapy, mammography screening rates, and the decline in breast cancer incidence specifically for Marin County, California, a population with historically elevated breast cancer incidence rates.

**Methods:**

The Marin Women's Study (MWS) is a community-based, prospective cohort study launched in 2006 to monitor changes in breast cancer, breast density, and personal and biologic risk factors among women living in Marin County. The MWS enrolled 1,833 women following routine screening mammography between October 2006 and July 2007. Participants completed a self-administered questionnaire that included items regarding historical hormone therapy regimen (estrogen only, progesterone only, EPHT), age of first and last use, total years of use, and reason(s) for stopping, as well as information regarding complementary hormone use. Questionnaire items were analyzed for 1,083 non-Hispanic white participants ages 50 and over. Breast cancer incidence rates were assessed overall and by tumor histology and estrogen receptor (ER) status for the years 1990-2007 using data from the Northern California Surveillance, Epidemiology and End Results (SEER) cancer registry.

**Results:**

Prevalence of EPHT use among non-Hispanic white women ages 50 and over declined sharply from 21.2% in 1998 to 6.7% by 2006-07. Estrogen only use declined from 26.9% in 1998 to 22.4% by 2006-07. Invasive breast cancer incidence rates declined 33.4% between 2001 and 2004, with drops most pronounced for ER+ cancers. These rate reductions corresponded to declines of about 50 cases per year, consistent with population attributable fraction estimates for EPHT-related breast cancer. Self-reported screening mammography rates did not change during this period. Use of alternative or complementary agents did not differ significantly between ever and never hormone users. Of women who reported stopping EPHT in the past 5 years, 60% cited "health risks" or "news reports" as their primary reasons for quitting.

**Conclusion:**

A dramatic reduction in EPHT use was followed temporally by a significant reduction in invasive and ER+ breast cancer rates among women living in Marin County, California.

## Background

The incidence of invasive breast cancer among non-Hispanic white women declined in the early part of this decade after a long period of increase [1-7]. Investigators surmised that the decrease could be due to one or more of several factors: decreases in use of combination estrogen-progestin menopausal hormone therapy (EPHT), treatment of in situ cancers, and saturation or decline in mammography screening. Although changes in mammography utilization could account for some of the decrease in incidence at the population level, sharp declines have been noted among populations subjected to uniform mammographic screening, indicating that the decline at the population level is unlikely to be due entirely to a change in mammography and is more likely to be explained by declines in EPHT utilization [8]. Large-scale reductions in utilization of EPHT are thought to have occurred after reports of negative health effects of EPHT from the Heart and Estrogen/Progestin Replacement Study (HERS) [9] and Women's Health Initiative (WHI) [10].

Marin County, California has received media and research attention due to high breast cancer rates in the mid- to late- 1990s. Earlier reports indicated that Marin County's incidence rates were 28% higher than other San Francisco Bay Area counties and 38% higher than other urban California counties combined [11]. In a case-control study of breast cancers conducted in 1997-99 in Marin County, Wrensch et al. [12] found no difference in ever use of EPHT in breast cancer cases compared to controls (35.4% vs. 36%, respectively). After the excess breast cancer incidence in Marin County was shown to be due to a disproportionate increase in estrogen receptor (ER)-positive breast cancers [13], a subsequent analysis of the same case-control study revealed that the excess of ER-positive breast cancers could, in part, be related to EPHT [14]. A recent assessment of EPHT usage in 2001 in 41 California counties utilizing data from the California Health Interview Survey (CHIS) [15], showed that Marin County ranked fourth among California counties with respect to EPHT use prevalence (estimated at 20.4% of all non-Hispanic white women aged 40-79), while overall hormone therapy use in Marin women was not higher than average usage in California. In light of the recent attention to changes in breast cancer and EPHT use, this report summarizes recent trends in breast cancer incidence, mammography screening rates, as well as in hormone therapy use and patterns of discontinuation, in Marin County.

## Methods

### Marin Women's Study

We used data from the Marin Women's Study (MWS), an ongoing prospective cohort study of women in Marin County funded by the CDC to examine breast cancer, breast density, and associated personal and biologic risk factors. The MWS was initiated in 2006 and is approved by the Marin General Hospital IRB. Women with Marin County residences are recruited at mammogram visits at each of six mammography centers in Marin County and at two hospitals in San Francisco that have substantial attendance from Marin County residents. Women scheduled to receive a mammogram are mailed, and asked to return, an enrollment packet (invitation letter, informed consent and releases, and scannable questionnaire). The packet is included with pre-registration materials required by the mammography clinic, but participation in the MWS is voluntary. Approximately 31% of the total population of Marin women are currently participating in the study.

An 87-item questionnaire was developed for use in the MWS, and assesses known and suspected breast cancer risk factors, using validated items where possible. The draft questionnaire was finalized in an iterative fashion such that initial items were pretested for acceptance and understanding in groups of 5-10 women, revised based on pretest results, pretested in another set of individuals, and revised again until the pretest results indicated no further revision was necessary.

### Study Population for Current Analysis

The current analysis is a retrospective examination of prior hormone use using a study population drawn from the MWS cohort. For this analysis, we included 1,833 women who attended mammography screening at Marin General Hospital, one of the larger participating clinics, between October 2006 and July 2007. Women were excluded if their mammogram was not a routine screening mammogram (n = 68), were younger than age 50 (n = 494), were missing data on age (n = 17), or were not non-Hispanic white race (n = 171) leaving an analysis population of 1,083.

### Breast Cancer Risk Factor Data

#### Menopausal Hormone Therapy

Women were asked to provide detailed information about their historical use of estrogen without progesterone (pills/patches alone, vaginal estrogen, and estrogen shots) and progesterone without estrogen (creams and pills/patches). Respondents were asked "Have you ever used prescription hormone replacement therapy (HRT) for symptoms of menopause or for other reasons?" To ascertain type of estrogen used, additional follow-up questions were asked, and included: "Have you ever used estrogen pills or patch alone (such as Premarin)?" "Have you ever used vaginal estrogen?" "Have you ever used estrogen shots?" "Have you ever used progestin cream?" "Have you ever used progestin pills or patch alone?" For each type of estrogen or progestin only regimen, women were asked the age when treatment was started and stopped and the total number of years the regimen was used.

In addition, women were asked about their current and past use of EPHT regimens. Women were asked "Have you ever used combination estrogen and progestin (such as Prempro, Premphase, or Premarin with Progestin)?" For each regimen, women were asked to indicate the type of estrogen and number of days a month they used estrogen, the type of progestin and the number of days they used progestin, their age when the regimen was started and stopped, the total number of years taken and the reason the regimen was discontinued (when applicable). Women were asked to document their most recent, or current regimen first. Response boxes were also provided for women to provide the same information on two previous regimens, if applicable.

#### Complementary and Alternative Medicines

Complementary and alternative medicine (CAM) utilization was ascertained for current use and use in the past 5 years. Stock at local pharmacies was reviewed to ascertain which CAMs to include as response options on the survey. Women were specifically queried about their use of combination herbal remedies (eg. Estroven and Estrohealth), black cohosh, chaste tree or berry, Dong Quai, phytoestrogen/plant estrogens, soy or soy supplements, wild yam and other. Women were asked to estimate the total length of time they used these supplements and the main reason for using it. Specific questions include: "During the past 5 years, have you used any of the following natural hormone supplements at least weekly for 3 or more months in a row?" "Please estimate the total length of time you have used these natural hormone supplements." "What was the main reason you used the natural hormone supplements?"

Based on self-reported start and stop date, yearly prevalence was calculated for estrogen therapy, EPHT and CAMS for single years between 1995 and 2006.

### Population Mammography and Hysterectomy Data

Mammography screening and hysterectomy rates for Marin County were ascertained from the California Health Interview Survey (CHIS) [16,17], using the CHIS 2001 and 2005 Adult Public Use Files. CHIS is a random-digit dial telephone survey of households in every county in California. CHIS covers a wide range of topics, including health status, health conditions, health-related behaviors, health insurance coverage, and access to and use of health care services. Respondents were asked: "Have you EVER had a mammogram?", and if yes, asked "How long ago did you have your most recent mammogram?", and "Have you ever had a hysterectomy?"

### Cancer Incidence Data

Annual cancer incidence and population data from 1990-2007 for Marin County and California were obtained from the Northern California Cancer Center, a part of the National Cancer Institute's Surveillance, Epidemiology, and End Results (SEER) Program. Analyses included new cases of invasive breast cancer using International Classification of Diseases - Oncology, 2nd edition, site codes 50.0-50.9 excluding histology codes 9590-9989 among non-Hispanic white women aged 50 years or older. For the purposes of this report and consistent with earlier reports documenting a particular increase in lobular cancer for Marin County [13], all invasive breast cancers were subdivided for stratified analysis into the following hormonal and histologic groups: estrogen receptor positive (ER+), estrogen receptor negative (ER-), lobular cases (ICD-0-3 morphology codes 8520 and 8522) and non-lobular/ductal cases (all other morphology codes, including unknown histology, but primarily consisting of code 8500/ductal histology).

### Statistical Analyses

Stata 8 (Stata Corporation) was used for all analyses of MWS data. Prevalence estimates and their 95% confidence intervals were calculated for each HT regimen for the current year (i.e., at the time of the survey in 2006/2007) and for prior years using recalled data. For years prior to the survey year (2006/2007), prevalence was calculated for women who were aged 50+ in that year (i.e., the denominator for each year only included women aged 50+ in that year). Statistical comparisons of the proportion of women using hormone therapy across years were made by comparing the 95% confidence intervals. A chi-squared statistic and the associated p-value were used to compare the reasons for quitting hormone therapy for those who quit within the past 5 years compared to those who quit 10 or more years ago. The online tool "AskCHIS" was used to calculate the prevalence of mammography use in 2001 and 2005 with 95% confidence intervals, as well as the prevalence of hysterectomy in 2005. SEER*Stat software (version 6.4.4) was used to calculate all age-adjusted and age-specific cancer incidence rates (age-adjusted to the 2000 US population standard), associated standard errors and 95% confidence intervals. Age-adjusted incidence rates were compared statistically using a Wald chi-square test of the difference between two rates with p-values of less than 0.05 considered significantly different. Use of Joinpoint regression was explored in preliminary analyses but yearly case counts were insufficient to detect year to year differences.

## Results

### Demographic Characteristics

Table 1 describes the demographic characteristics of the study population compared to estimates for Marin County as a whole. The average age of the 1,083 non-Hispanic white women included in this analysis was 62.5, ranging from 50-95, which is similar to Marin overall. There was broad representation of income groups in this study population, although compared to Marin as a whole, a lower percentage of women in the lowest income and education groups are represented in the MWS study population. As with the general Marin County population, the study population was fairly well educated, with 36.0% having obtained a college degree, and an additional 31.1% having obtained a post-baccalaureate degree. The majority of the study population was married (63.6%), with an additional 25.9% being currently single but having been married in the past. Eighteen percent of the MWS sample had a hysterectomy, compared to 23.1% of non-Hispanic white women ages 50+ in Marin [16].

**Table 1:** Demographic characteristics, non-Hispanic white women ages 50 and over, Marin Women's Study 2006-2007 (n = 1083) and Marin County as a whole.

	n	MWS2006-07 %	CHIS 2005-07 %
**Age**			
Mean		62.5	64.5
Range		50-95	50-96
Age Categories			
50-59	482	44.5	41.3
60-69	372	34.4	27.8
70-79	158	14.6	18.4
80+	71	6.6	12.5
			
**Education**			
H.S or less	62	5.7	17.6
Some college/AA	281	26.0	24.1
BS	390	36.0	35.7
MS	246	22.7	17.5
Doctoral/Professional	91	8.4	5.2
DK/Missing	13	1.2	-
			
**Marital Status**			
Married	689	63.6	49.9
Widowed/Divorced/Separated	280	25.9	37.2
Single	68	6.3	8.6
Living Together	44	4.1	4.3
Missing	2	0.2	-
			
**Income**			
<$75,000	219	20.2	52.3
$75-$150,000	354	32.7	33.4
>$150,000	341	31.5	14.3
Declined/Don't know	169	1.3	-
Missing	2	14.3	-
			
**Ever Hysterectomy**			
Yes	195	18.0	23.1
No	888	82.0	76.9

### Postmenopausal Hormone Therapy Use

#### Ever Use

Table 2 shows the various hormone therapy regimens reported as being used by this study population between 1995 and 2006/2007. The percentage of women ages 50 or older in 2006/2007 who reported ever using EPHT, EHT only, and/or progestin pill/patch was 56.6% overall, with 25.3% reporting ever using EPHT, 39.9% reporting ever use of EHT alone, and 5.9% reporting ever use of a progestin only pill or patch.

**Table 2:** Hormone therapy usage, non-Hispanic white women ages 50 and over, Marin Women's Study 2006/2007 (n = 1083)

		n	%
**Ever Use -- prescription hormone therapy**		613*	56.6
Estrogen Only	Ever	432	39.9
	Never	625	57.7
	Missing	26	2.4
			
EPHT	Ever	274	25.3
	Never	809	74.7
	Missing	0	0
			
Progesterone	Ever	64	5.9
Pill/Patch	Never	1008	93.1
	Missing	11	1.0
			
**Ever Use -- prescription hormone therapy by age category**			
	50-59	226	33.0
	60-69	291	42.5
	70-79	124	18.1
	80+	44	6.4
	missing	0	0
			
**Current Use -- prescription hormone therapy**		282*	26.0
Estrogen Only	No	840	77.6
	Yes	243	22.4
	Missing	0	0
**Type**			
Pills/Patch	No	967	89.3
	Yes	110	10.2
	Missing	6	0.6
			
Vaginal Estrogen	No	941	86.9
	Yes	142	13.1
	Missing	0	0
			
EPHT**	No	1010	93.3
	Yes	72	6.7
	Missing	1	0.1
			
Progesterone	No	1062	98.1
Pill/Patch	Yes	20	1.9
	Missing	1	0.1
			
**EPHT Use by Age and Year**		**2001 (%, 95% CI)**	**2006 (%, 95% CI)**
	50-59	21.9 (18.2, 25.7)	10.6 (7.8, 13.2)
	60-69	21.2 (15.8, 26.6)	9.7 (6.6, 12.8)
	70-79	5.6 (1.2, 10.0)	4.8 (1.3, 8.4)
	80+	0	0

*Does not represent simple sum as regimens are not mutually exclusive

**Estrogen plus progestin hormone therapy

#### Current Use

The proportion of women currently taking prescription hormone therapy in 2006/2007 in this study population was 26.0%, with 6.7% of women reporting current EPHT use, 22.4% reporting EHT only, and 1.9% progestin only.

#### Trends

**Figure 1:**
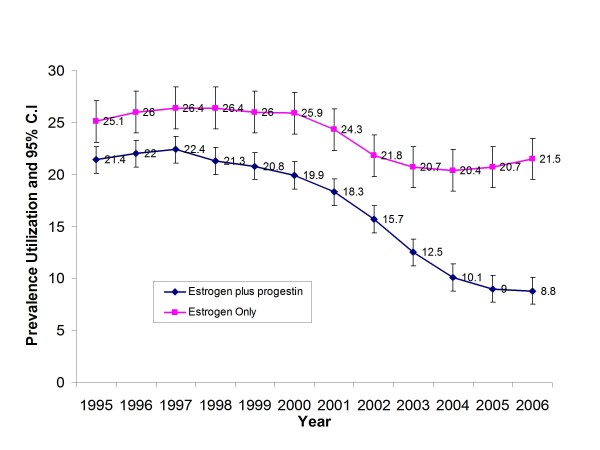
**Trends in combination estrogen plus progestin and estrogen only hormone therapy use among non-Hispanic white women, ages 50 and over in Marin County 1995-2006**.

Figure 1 illustrates the prevalence of EHT and EPHT utilization among women aged 50 and older from 1995 to 2006. Use of EHT decreased 15.4% over the time period, though year-to-year decreases were not statistically significant. In contrast, use of EPHT changed dramatically from 1995 to 2006. Overall, a 58.9% reduction was observed during that time period. The proportion of women currently using EPHT and the proportion using it in 2006 were significantly lower than the proportion using it in every year prior to 2004.

We examined the relative decreases in EPHT use taking into consideration the publication of the HERS study in 1998 and the WHI trial in 2002. From 1995 to 1998, EPHT use was steady (0% overall change). Between publication of the HERS and WHI trials (i.e., 1998 to 2001), there was a 14% decrease in EPHT use. The largest decrease occurred after publication of the WHI trial data in 2002. From 2002 to the time of the survey (2006/2007), there was a 57.3% drop in EPHT use.

To understand whether overall changes in EPHT use stemmed from decreases in use for particular age groups, prevalence of use by age group was calculated for the years 2001 and 2006 (Table 2). While use in 2006 was lower than that in 2001 in women 50-59 and 60-69, there was no evidence of changes in use among women aged 70 years and older.

#### Initiation and Cessation Behavior

The timing of initiation and cessation of HT is presented in Table 3. 51.6% of prior EPHT users quit within the past 5 years, which was slightly lower for EHT users (43.8%). Of current HT users, 47.2% of EPHT users started taking EPHT within the past 5 years (after WHI and HERS), compared to 53% of current EHT users.

**Table 3:** Initiation and cessation of prescription hormone therapy by regimen, non-Hispanic white women ages 50 and over in Marin County, 2006-2007

Regimen	Utilization Patterns	n	%	
**Estrogen Only (EHT)**	Past Users who **quit **within past 5 years	91	43.8 of past users	
	Ever Users who **started **in past 5 years	148	34.2	
	Current users, time started:			
	≤5 years ago	115	53.0	
	> 5-10 years ago	47	21.7	
	> 10 years ago	55	25.4	
				
**Estrogen plus progestin (EPHT)**	Past Users who **Quit **within past 5 years	100	51.6 of past users	
	Ever Users who **started **in past 5 years	53	19.3	
	Current users, time started:			
	≤5 years ago	34	47.2	
	> 5-10 years ago	17	23.6	
	> 10 years ago	21	29.2	
				
	**Reasons quit (past users)**	**Quit < 5 yrs ago**	**Quit >10 yrs ago**	**X ^2^, p-value**
		**n (%)**	**n (%)**	
	Side Effects	11 (11.0)	9 (33.3)	7.99, 0.01
	Doctor Recommended	32 (32.0)	7 (25.9)	0.37, 0.54
	Health Effects	38 (38.0)	6 (22.2)	2.34, 0.13
	News Reports	47 (47.0)	4 (14.8)	9.16, 0.002
	Switched types	6 (6.0)	2 (7.4)	0.07, 0.79
	Health or News Reports	60 (60.0)	8 (29.6)	7.89, 0.01

The reasons prior users reported stopping EPHT are also shown in Table 3. (These data were not gathered for EHT use.) The proportion of women who cite quitting EPHT due to health effects and/or news reports is significantly greater among those who quit within the past 5 years than among those who quit 10 or more years ago (60.0% vs. 29.6%, respectively). The proportion of those who quit because of a doctor's recommendation was non-significantly higher among those who quit within the past 5 years compared to those who quit 10 or more years ago (32.0% vs. 25.9%), while the proportion citing side effects is significantly lower (11.0% vs. 33.3%, respectively).

#### Complementary & Alternative Medicines

In this study population, 9.3% of women reported currently using CAMs for the symptoms of menopause (Table 4), which is slightly higher than current EPHT usage (6.7% of women). The prevalence of current use of CAMs did not vary by history of HT utilization. Soy products, black cohosh, and combination herbal remedies were the CAMs most commonly reported as being currently used (2.5%, 2.3%, and 1.4% of women using, respectively).

**Table 4:** Prevalence of complementary and alternative medicine (CAM) usage for menopausal symptoms, non-Hispanic white women ages 50 and over, Marin County, 2006-2007 (n = 1083)

	n	%
**Currently Using**	101	9.3
Soy Products	27	2.5
Phytoestrogens	8	0.7
Combination Herbal	18	1.4
Black Cohosh	25	2.3
Chaste Tree	6	0.6
Wild Yam	6	0.6
Other	19	1.8
Progestin Cream	21	1.9
		
**Current Users, length of time**		
<1 year	37	34.6
1 - 2 years	27	25.2
3 - 4 years	19	17.8
5+ years	24	22.4
**CAM use in women who have ever used hormone therapy**	57	9.6
**CAM use in women who have never used hormone therapy**	44	9.0

### Invasive Breast Cancer Incidence Rates

**Figure 2:**
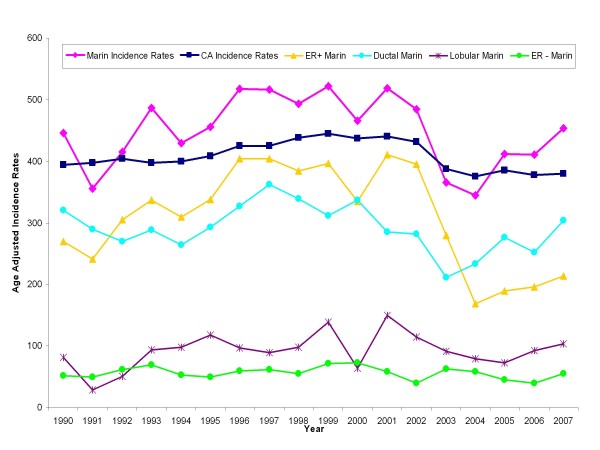
**Trends in age-adjusted invasive breast cancer incidence rates, non-Hispanic white women ages 50 and over, Marin County and California**. ER+, ER-, ductal and lobular component rates Marin County, 1990-2007.

Figure 2 and Table 5 show annual age-adjusted invasive breast cancer incidence rates from 1990 to 2007 for non-Hispanic white women, aged 50 years and older living in Marin County at the time of diagnosis. Rates reached their absolute peak of 522.2/100,000 (CI 452.9-599.2) in 1999, an increase of 17% from the beginning of the period examined (1990). Rates were lowest in 2004, at 345.1 cases/100,000 (CI 291.8-406.3). By the end of the period examined (2007), the incidence rate in Marin County had increased to 453.5/100,000 (CI 392.1-522.7), which was significantly higher than the corresponding rate for the state of California 379.3/100,000 (CI 372.4-386.2), but was not significantly higher than the lowest rate for Marin in 2004.

**Table 5:** Case counts and age adjusted incidence rates: invasive breast cancer, ER+, ER-, ductal and lobular component, non-Hispanic white women 50+ Marin County, 1990-2007

	Invasive Breast Cancer			ER+			ER-		
	Count	Rate	95% C.I.	Count	Rate	95% C.I.	Count	Rate	95% C.I.
1990	143	445.4	(374.8 - 526.2)	86	269.6	(215.2 - 334.3)	16	51.3	(29.1 - 84.4)
1991	119	356.1	(294.7 - 427.2)	81	241.2	(191.3 - 300.8)	16	49.3	(28.0 - 81.1)
1992	138	415.3	(348.6 - 491.7)	102	305.5	(248.8 - 371.8)	20	62	(37.7 - 96.5)
1993	166	486.4	(415.0 - 567.1)	115	336.5	(277.6 - 404.6)	23	69.8	(44.2 - 105.2)
1994	150	429.5	(363.3 - 504.6)	108	309.7	(253.9 - 374.5)	18	52.7	(31.2 - 83.8)
1995	160	456.1	(387.9 - 533.0)	118	338.3	(279.8 - 405.6)	17	49.8	(29.0 - 80.0)
1996	184	517.5	(445.2 - 598.5)	144	404	(340.5 - 476.2)	21	59.6	(36.9 - 91.6)
1997	191	516	(445.1 - 595.2)	148	403.7	(341.1 - 474.8)	23	61.8	(39.1 - 93.3)
1998	187	492.7	(424.3 - 569.3)	144	384.1	(323.7 - 452.7)	21	55.1	(34.1 - 84.8)
1999	208	522	(452.9 - 599.2)	157	396.1	(336.1 - 464.2)	29	71.3	(47.6 - 103.5)
2000	188	466	(401.1 - 538.9)	132	335	(279.8 - 398.4)	30	73	(49.0 - 105.3)
2001	210	518	(449.5 - 594.5)	165	410.8	(349.8 - 479.9)	24	58.2	(37.1 - 87.7)
2002	200	484.6	(418.9 - 558.3)	163	395.3	(336.2 - 462.5)	16	39.7	(22.5 - 65.6)
2003	156	365	(309.1 - 428.8)	120	280.1	(231.5 - 336.6)	26	62.3	(40.4 - 92.7)
2004	154	345.1	(291.8 - 406.3)	75	167.9	(131.5 - 212.3)	26	58.7	(38.0 - 87.9)
2005	183	411.7	(353.1 - 478.3)	86	189.4	(151.0 - 235.8)	19	44.6	(26.5 - 71.4)
2006	190	410.7	(353.1 - 476.0)	93	196.1	(157.4 - 242.6)	19	39.9	(23.8 - 64.2)
2007	205	453.5	(392.1 - 522.7)	98	213.7	(172.5 - 262.6)	25	55.1	(35.2 - 83.4)
									
	**Ductal**			**Lobular Component**					
	**Count**	**Rate**	**95% C.I.**	**Count**	**Rate**	**95% C.I.**			
1990	103	320.8	(261.3 - 390.4)	26	81	(52.7 - 120.0)			
1991	97	289.7	(234.6 - 354.5)	9	28.3	(12.9 - 54.7)			
1992	89	270.1	(216.7 - 333.3)	17	50.3	(29.2 - 81.5)			
1993	98	288.4	(234.0 - 352.2)	32	93.5	(63.9 - 132.8)			
1994	92	264.1	(212.7 - 324.5)	34	98.2	(67.9 - 137.7)			
1995	102	292.6	(238.4 - 355.7)	41	117.6	(84.3 - 159.9)			
1996	116	326.6	(269.7 - 392.2)	34	97.1	(67.1 - 136.1)			
1997	134	362.2	(303.2 - 429.6)	32	89.2	(61.0 - 126.3)			
1998	128	338.6	(282.3 - 403.2)	37	97.9	(68.8 - 135.5)			
1999	124	311.5	(258.7 - 372.5)	54	138.9	(104.1 - 182.2)			
2000	136	337.2	(282.4 - 400.1)	25	64.3	(41.4 - 96.0)			
2001	116	285.6	(235.5 - 344.0)	60	149.9	(114.0 - 194.2)			
2002	116	282.3	(232.6 - 340.1)	47	114	(83.3 - 152.8)			
2003	90	211.8	(169.7 - 262.0)	40	90.9	(64.6 - 125.4)			
2004	103	233	(189.3 - 284.7)	36	79.3	(55.3 - 111.5)			
2005	123	276.7	(229.0 - 332.3)	33	72.2	(49.4 - 103.4)			
2006	118	251.7	(207.5 - 303.7)	42	92.7	(66.2 - 127.5)			
2007	138	303.8	(254.1 - 361.2)	45	103.5	(74.9 - 140.3)			

**Figure 3:**
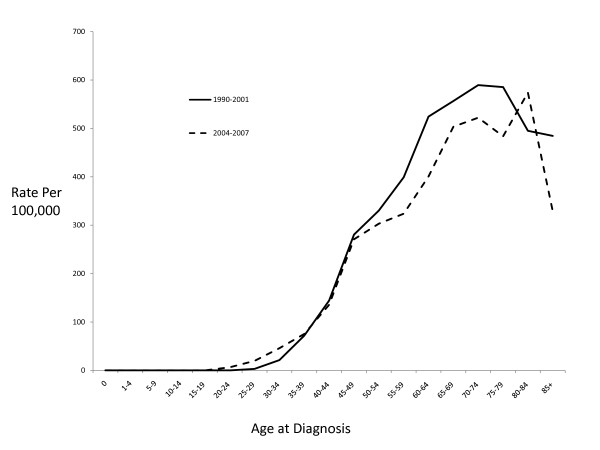
**Age-specific incidence rates of female invasive breast cancer among non-Hispanic white women in Marin County**.

Figure 3 shows age-specific incidence rates for two time periods, 1990-2001 and 2004-2007. Although the differences were not statistically significant, the change in incidence rates appears to be limited to women over the age of 50 and was greater for the 50-79 year age group than for women older than 80, although rates were somewhat unstable for the older age groups.

### Breast Cancer Histology and ER Status

The incidence of ER+ tumors in Marin remained relatively constant from 1990 though 2001; ER+ tumors then dropped sharply and significantly between 2002 and 2004, from 395.3/100,000 to 167.9/100,000 representing a reduction of over 57% (34.8% a year) or 88 incident cases (Figure 3). Despite small numbers of cases, there was no evidence of a significant change in ER- tumor incidence during that same time period. Case counts, age-adjusted incidence rates and corresponding confidence intervals are presented in Table 5.

Figure 2 also shows the incidence trends for invasive breast cancers according to histologic subtype. Between 1990 and 2007, 70-80% of all breast cancer cases in Marin County were of ductal/non-lobular histology. Ductal invasive carcinomas decreased significantly in Marin County from 362.2/100,000 (CI 303.2-429.6) in 1997 to a low of 211.8/100,000 (CI 169.7-262.0) in 2003, representing an 8.6% average annual reduction. Despite small numbers of cases for analysis, the pattern in lobular breast cancer incidence differed somewhat from that of ductal breast cancers. Rates of lobular cancers increased throughout the 1990s, with the rates in 1999 and 2001 significantly higher than 1991 and 1992. From 2001-2006, rates decreased somewhat, but these differences did not reach statistical significance.

### Prevalence of Mammography Screening

CHIS survey data suggested consistent trends in self-reported rates of biannual mammography screening in Marin County with prevalences of 87.2% (CI 81.7-92.7) in 2001 and 85% (CI 82.8-87.3) in 2005 [16,17]. In California, the prevalences were 82.7% (81.8-83.7) in 2001 and 83.9% (83.0-84.9) in 2005, not significantly different than the reported screening prevalence in Marin. Differentiation between first and subsequent mammograms could not be ascertained from this data source.

## Discussion and Conclusions

Invasive breast cancer incidence rates in Marin County among non-Hispanic white women aged 50 years or older decreased significantly between 2001 and 2004, a reduction of 33% (7.9% per year). The change in incidence rates was evident primarily in women ages 50-79. During the same time period, EPHT utilization dropped more than 50% (9.9% per year) in women 50 and older, and continued to drop through 2006, while mammography screening rates remained stable. The reduction in EPHT utilization preceded the drop in invasive breast cancer incidence rates, and was seen as early as 1998, with a more precipitous drop evident between 2001 and 2004.

Early in the investigation of high breast cancer rates in Marin County, we explored HT use as a possible reason for the high rates. In the absence of more specific data on prevalence of EPHT vs. EHT use, we found that overall HT use in Marin County women ages 50 and over was not significantly different than the prevalence for the entire state of California (CHIS 2001). Subsequent analyses using updated data that differentiated between HT formulations showed that Marin had the 4^th ^highest estimated EPHT prevalence among California counties [15]. One explanation for the high prevalence of EPHT utilization in the absence of excess overall HT use is a difference in the prevalence of hysterectomy. Indeed, further investigation for this manuscript revealed substantial county by county variations in hysterectomy rates throughout California, ranging from 14.9% to 55.8% (San Francisco and Sutter Counties, respectively) in white women ages 50+, with Marin having the second lowest percentage of hysterectomies (23.1%) [17]. Understanding why hysterectomies vary so widely geographically, while not the subject of this manuscript, warrants further investigation.

While our survey did not specifically query women about HERS and WHI and the resulting press coverage, we believe that the reduction in HT use is largely attributable to the publication and subsequent dissemination of these results, as has been documented in other populations. Interestingly, the proportions of women reporting physician-initiated cessation of EPHT did not increase in the post-WHI period (Table 3). This may relate to the high educational status and good media access of the study population in question, which may lead women to discontinue treatment on their own following negative press on EPHT.

Complementary and alternative hormone (CAM) utilization did not significantly increase despite the fact that EPHT and EHT use dropped precipitously, suggesting that women are not replacing prescription hormone therapies with CAMs as a treatment for menopausal symptoms. Future research should address whether women are seeking other therapies to replace EPHT for the treatment of menopausal symptoms, and should address the relative safety and efficacy of these therapies.

Previous assessments in Marin County women suggested that the excess breast cancer incidence relative to other urban California geographic areas was predominantly ER+ lobular and ductal breast cancer cases [13]. Total ER+ Marin breast cancer cases diagnosed between 1997-1998 were associated with excess postmenopausal hormone therapy use [14]. Larger population studies of breast cancer subtypes associated with current and long duration EPHT use have been somewhat contradictory [18], but in general suggest a link between EPHT use and increased ER+ breast cancer incidence [5,13,18]. In the present study, the changing incidence of lobular and ductal breast cancer were not concordant with one another between 1999 and 2003, and changes in total invasive breast cancer incidence over this period largely reflected that of the more commonly diagnosed ductal subtype of breast cancer. Almost all lobular breast cancers and over 60% of ductal breast cancers are ER+; therefore, it is noteworthy that the change in total ER+ breast cancer incidence rates between 1999 and 2003 not only paralleled the change in total invasive breast cancer incidence but showed a precipitous decline after 2001 that better reflects the change in EPHT use than did the decline in ductal breast cancer rates. Following the decline in EPHT use between 2000-2004, ER+ breast cancer incidence rates dropped by >50% while ductal breast cancer incidence dropped by only 29% and lobular rates showed no significant change, but the number of lobular cancers is small making changes in these rates difficult to detect (Figure 2).

To understand whether the observed reduction in EPHT therapy was commensurate with the drop in breast cancer cases seen in Marin County, we referred to a recent analysis of the population-level impact of HT on breast cancer rates which used the population attributable fraction (PAF) to estimate the proportion of breast cancer incidence rates attributable to EPHT [19]. PAF estimates were calculated based on a range of EPHT use (5-22%) and relative risk estimates from the literature (1.24-3.06) for the risk of EPHT on breast cancer. When PAF estimates are calculated in the MWS study population, using an average prevalence of EPHT use (20%) from 1999-2001 and moderate relative risk estimates of 1.5 - 2.0, these PAF estimates would suggest that approximately 22 - 40 cases annually (10-18% of 217 invasive breast cancer cases per year in 2001-2002 in Marin) could be attributable to EPHT use in the years before 2002, commensurate with our observations of 50 fewer cases per year in Marin County.

The drop in invasive breast cancer incidence in 2002-2004 was followed by a non-significant increase in incidence rates in 2005-2007. Determining whether this is a real increase in incidence will only be possible with additional years' data. The increase may be a statistical aberration given the relatively small number of annual cases in Marin (and therefore unstable rates), or could be due to inaccuracies in the population estimates [20]. On the other hand, if the rate increase proves to be real, it could be due to replacement of EPHT by another compound also associated with breast cancer, a rebound emergence of cases after some delay due to promoting influences that only temporarily retarded the emergence of new tumor cases, improved sensitivity of mammography after removal of EPHT which impeded detection of breast cancer [21], or a reflection of downward resetting of the Marin incidence curve that reveals another underlying increasing incidence trend that has been happening independent of EPHT. It will be important to continue monitoring breast cancer incidence rates, the prevalence of HT and other risk factors, as well as mammography screening changes to determine whether this trend continues.

Mammography screening rates in Marin County, as assessed by a statewide population-based survey [16,17], decreased non-significantly during the study period, suggesting that a large drop-off in mammography utilization does not explain the breast cancer rate decline in this population. Similarly, in California, reported screening rates did not change significantly during this time period and were not significantly different from those in Marin. Although overall screening rates did not change significantly, a screening saturation effect should also be considered in assessing changes in incidence rates. Specifically, the proportion of mammography-detected cancers that are interval cancers versus existing previously-undiagnosed cancers will vary depending on the extent of screening saturation in the population. A change in the distribution of first to subsequent mammograms (whether cases were detected at first screen, subsequent screen or not screen detected) and thus a change in screen-detected cases, has been shown to explain recent incidence trends outside the U.S. [22,23], but could not be determined in this study. While screening saturation prior to 2001 cannot be directly assessed in the Marin population using these data, one study reporting on screening patterns in the Western United States [5], indicates that saturation occurred in the late 1980s to early 1990s. It is not likely that Marin deviated substantially from this pattern. In addition, a recently published manuscript of a California cohort of women with similar demographics to Marin found invasive incidence rates declined significantly in 2003-2005 with consistent levels of screening throughout the study period [24]. Given the high prevalence of EPHT utilization and the substantial, subsequent decline in use in this population, changes in screen-detected cases would need to be considerable during this time period to explain such a dramatic drop in breast cancer rates in Marin, although the possibility cannot be discounted. Future research must continue to monitor mammography utilization patterns alongside EPHT utilization to examine any association with future changes in breast cancer incidence rates.

While this interim analysis of the MWS has a number of strengths, including a large sample size and in-depth individual-level data on use of EPHT and EHT, as well as the reasons for stopping EPHT, there are a number of limitations to this study. The primary limitation is the possibility of selection bias. We were only able to obtain information from women currently living in Marin County, thus excluding women who lived in Marin County during the retrospective study period but who are not available for interview due to out-migration or death. It has been hypothesized that migration of low or high risk women on the basis of changes in the cost of living during the 1990's could have affected geographic patterns in breast cancer incidence [11,25]. Given the association between EPHT utilization and adverse outcomes, it is possible that our estimates of EPHT use are lower than the true prevalences, as current users may have been more likely to die and thus not be a part of the current study. In addition, the potential for selection bias is also present in the form of preferential participation by women who perceive themselves to be at increased risk of breast cancer. However, our estimates of the prevalences of HT utilization in this study are similar to those published previously [26], indicating a lack of a strong selection bias.

HT utilization was assessed in this study in a population with uniform access and utilization of mammographic screening, suggesting a potential source of selection bias. As expected, given that this is service-based population, women with lower SES who have less access to care are not well represented the MWS study population. With the exception of the lowest income and education groups, women in the mammography screening population are seen as being representative of the broader population of Marin County women ages 50 and over, as 70% of women in this age group in Marin report having a mammogram in the past year [27] and approximately 90% report having had a mammogram in the past two years [28]. In addition, the clinic at Marin General Hospital serves clients with both HMO and PPO insurance plans and has referrals from local community clinics for the uninsured to receive low- and no-cost mammography visits. Efforts continue to include low SES women in ongoing MWS recruitment. Though the income distribution may seem to indicate that only high income women are included in the study population, the cost of living in Marin County is very high, thus, these incomes represent the range of incomes found in this area.

A second limitation in the current study is the potential for information bias in the ascertainment of HT utilization. HT utilization was based on self-report, rather than clinical records, and is therefore subject to problems in recall. Again, we do not see this as a major problem in the current study as the estimated prevalence of HT use in 2001 in this study population was similar to that found in two cross-sectional health surveys [16,17] conducted in Marin County in 2001. In addition, a recent study by Banks et al. [29], compared medical practice prescription records to self-reported questionnaire data from women participating in the Million Women Study and found excellent agreement between the two.

Another potential source of information bias is in the calculation of breast cancer incidence rates. Cancer incidence trends can be biased by inaccurate cancer registry or population projections if the projections or registry errors change over time. The California Cancer Registry has produced extremely accurate case counts; case ascertainment was estimated at 99% for 2004 [30]. Intercensal population projections, on the other hand, can be substantively incorrect, and have biased breast cancer incidence rate estimation in Marin County in the past decade [20]. We will not be able to ascertain the extent to which bias may be present in the incidence rates until the 2010 Census is conducted.

This study was not able to include a range of potentially confounding risk factors known to play a role in breast cancer incidence rates, as comprehensive risk factor information in the study population were not available at the time of publication. We presented this ecologic, retrospective analysis of breast cancer incidence in relation to hormone therapy use, similar to other published reports [4,6,8,15] which assessed changes in EPHT use after publication of the WHI results. Additional risk factors and their relationship to breast cancer incidence will be the subject of future reports, although current nationwide trends in BMI (increasing), parity (decreasing), and late childbearing (increasing) should tend to increase breast cancer rates if those trends are mirrored in Marin County. We believe it is unlikely that family history of breast cancer changed significantly during this time period to impact breast cancer rates.

Although Marin County is a unique population in terms of education and income, Marin has been a bellwether for cancer trends in the past. In the late 1970's Marin's uterine cancer rates spiked precipitously, providing strong evidence for the potential risks of EHT on the uterus. Thus, despite its distinctive demographics, the relationship between etiologic factors and cancer are not unique to Marin, and are applicable to women across the world. Studying populations with extreme risk factor prevalences can help to signal risk that may be more difficult to study in populations with more moderate prevalence. While the proportion of women using EPHT may not have dipped as low in other populations as it has in Marin County, it has decreased as have breast cancer rates, so the trends and underlying relationship are not unique to Marin County.

In this study we were able to examine breast cancer incidence and the pattern of HT utilization and cessation during a period of dramatically altered views on the health benefits of HT. The extent to which women quit using EPHT and the lower adoption of EPHT in the years following the publication of the HERS and WHI study results is consistent with other reports from elsewhere in California and the US, as was the subsequent decline in breast cancer incidence, particularly ER+ breast cancers, and the estimated proportions of Marin County breast cancers attributable to current EPHT use. Future studies addressing the predictors of HT utilization in the post-WHI era will enhance these results by determining which populations are most affected by this changing behavior, and will therefore be most likely to experience changes in breast cancer incidence. Continued attention to population-based patterns of HT utilization and breast cancer incidence will reveal whether the HERS and WHI trials resulted in a sustained, long-term reduction in the incidence of HT-related breast cancers.

## Abbreviations

EPHT: Estrogen plus Progestin Menopausal Hormone Therapy; MWS: Marin Women's Study; ER: Estrogen Receptor; CHIS: California Health Interview Survey; CDC: Centers for Disease Control; IRB: Internal Review Board; HRT: Hormone Replacement Therapy; CAM: Complementary and Alternative Medicine; RDD: Random-Digit Dial; SEER: National Cancer Institute's Surveillance, Epidemiology, and End Results; EHT: Estrogen Hormone Therapy; HERS: Heart and Estrogen/progestin Replacement Study; WHI: Women's Health Initiative; HT: Hormone Therapy; PAF: Population Attributable Fraction; SES: Socio-Economic Status.

## Competing interests

CAC has served as an expert  witness for plaintiff lawyers preparing hormone therapy litigation.  

## Authors' contributions

All authors contributed to the data collection and design of the Marin Women's Study. RE conceived of the study, LP analyzed the MWS data, CAC analyzed the breast cancer incidence data, RE and LP wrote the manuscript, CAC and CB collaborated on writing the manuscript. All authors have read and approved the final manuscript.

## Pre-publication history

The pre-publication history for this paper can be accessed here:

http://www.biomedcentral.com/1471-2458/10/228/prepub
